# Formation of Cytoplasmic Actin-Cofilin Rods is Triggered by Metabolic Stress and Changes in Cellular pH

**DOI:** 10.3389/fcell.2021.742310

**Published:** 2021-11-17

**Authors:** Hellen C. Ishikawa-Ankerhold, Sophie Kurzbach, Arzu S. Kinali, Annette Müller-Taubenberger

**Affiliations:** ^1^ Department of Internal Medicine I, University Hospital, LMU Munich, Munich, Germany; ^2^ Walter Brendel Centre of Experimental Medicine, University Hospital, LMU Munich, Munich, Germany; ^3^ Department of Cell Biology (Anatomy III), Biomedical Center (BMC), LMU Munich, Munich, Germany

**Keywords:** actin, cofilin, cytoskeleton, cytoplasmic rod, *Dictyostelium discoideum*, intracellular pH, metabolic stress, neurodegenerative disease

## Abstract

Actin dynamics plays a crucial role in regulating essential cell functions and thereby is largely responsible to a considerable extent for cellular energy consumption. Certain pathological conditions in humans, like neurological disorders such as Alzheimer’s disease or amyotrophic lateral sclerosis (ALS) as well as variants of nemaline myopathy are associated with cytoskeletal abnormalities, so-called actin-cofilin rods. Actin-cofilin rods are aggregates consisting mainly of actin and cofilin, which are formed as a result of cellular stress and thereby help to ensure the survival of cells under unfavorable conditions. We have used *Dictyostelium discoideum*, an established model system for cytoskeletal research to study formation and principles of cytoplasmic actin rod assembly in response to energy depletion. Experimentally, depletion of ATP was provoked by addition of either sodium azide, dinitrophenol, or 2-deoxy-glucose, and the formation of rod assembly was recorded by live-cell imaging. Furthermore, we show that hyperosmotic shock induces actin-cofilin rods, and that a drop in the intracellular pH accompanies this condition. Our data reveal that acidification of the cytoplasm can induce the formation of actin-cofilin rods to varying degrees and suggest that a local reduction in cellular pH may be a cause for the formation of cytoplasmic rods. We hypothesize that local phase separation mechanistically triggers the assembly of actin-cofilin rods and thereby influences the material properties of actin structures.

## Introduction

Actin is among the most abundant and most highly conserved proteins in eukaryotic cells, and as such essential for many processes including cell growth, differentiation, cell division, motility, intracellular movement, and mechanic stability. To fulfil these different functions, actin assemblies form a variety of dynamically regulated membrane-associated or intracellular structures (Chhabra and Higgs, 2007), and after long debates on the existence of actin in the nucleus, the specific roles have been elucidated in recent years (Kelpsch and Tootle, 2018; Caridi et al., 2019; Hyrskyluoto and Vartiainen, 2020).

Actin serves as both a structural and a force-generating protein. Inside cells, there is a dynamic equilibrium of monomeric actin (G-actin) sequestered by monomer-binding proteins like profilin, and filamentous actin (F-actin). Monomeric actin contains one nucleotide and can exist either in the ATP, ADP-Pi or ADP form. The assembly and disassembly of actin filaments is principally dependent upon the concentrations of actin monomers, and the critical concentrations for actin assembly vary as actin filaments themselves show a polarity and have different critical concentrations for polymerization on either end. The assembly of monomeric actin into filaments and the disassembly of filamentous actin involves a whole arsenal of actin-binding proteins that regulate actin functions including nucleation, sequestering, and crosslinking, and the highly energy-dependent dynamic turnover has been described as “actin-treadmilling” (Pollard and Borisy, 2003).

Among the proteins crucial for the dynamic turnover of actin filaments *in vivo* are those of the actin-depolymerizing factor (ADF)/cofilin family. Members of the cofilin family are highly conserved and functionally related proteins (Bernstein and Bamburg, 2010; Kanellos and Frame, 2016). Cofilin severs actin filaments at low cofilin/actin ratios and stabilizes filaments at high ratios. The activity of cofilin is enhanced by coronin 1A (Cai et al., 2007), which helps in recruiting cofilin, and actin-interacting protein 1 (Aip1), which enhances the severing function of cofilin (Pollard and Borisy, 2003; Kanellos and Frame, 2016). In contrast to mammalian cells, in *Dictyostelium*, cofilin activity is not regulated by phosphorylation (Aizawa et al., 1995). LIMK1/2 (or TESK1/2) and slingshot (SSH1L) phosphatase are not present. The absence of this regulation in *Dictyostelium* simplifies the approach to investigate the general principles of cofilin functions on actin organization under different conditions.

Several disease pathologies are characterized by the polymerization of actin into stable filament bundles called actin-cofilin rods. Actin-cofilin rods consist of equimolar ratios of actin and cofilin (Nishida et al., 1987; Minamide et al., 2010). Rods can be formed either inside the nucleus or the cytoplasm. Cytoplasmic rods are associated with several neurodegenerative diseases like Alzheimer’s and Parkinson’s disease, variants of amyotrophic lateral sclerosis (ALS) (Bamburg and Wiggan, 2002; Bamburg and Bernstein, 2010; Schonhofen et al., 2014; Bamburg and Bernstein, 2016; Wang et al., 2020), and spinal muscular atrophy (SMA) (Walter et al., 2021). Intranuclear actin-cofilin rods were identified as hallmarks in muscle cells of patients with intranuclear rod myopathy (IRM), a specific form of nemaline myopathy (Goebel and Warlo, 1997; Sparrow et al., 2003; Ilkovski et al., 2004; Domazetovska et al., 2007a; Domazetovska et al., 2007), and Huntington’s disease (HD) (Munsie et al., 2011). However, our understanding of the specific mechanisms that cause actin-cofilin rod formation in either the cytoplasm or the nucleus, and how rod formation is modulated by physiological parameters is still incomplete.

The *Dictyostelium discoideum* system has many technical advantages in comparison to mammalian models and pioneered a variety of scientific approaches to study basic cellular functions relevant for disease-related states (Müller-Taubenberger et al., 2013). In fact, before their description in neuronal cells, nuclear actin-cofilin rods were discovered in *Dictyostelium* amoebae and in HeLa cells after treatment with high concentrations of dimethyl sulfoxide (Fukui, 1978; Fukui and Katsumaru, 1979, 1980; Sanger et al., 1980). Previous work from our lab focused on the analysis and characterization of nuclear rods in *Dictyostelium* (Ishikawa-Ankerhold et al., 2017). Thereby, we examined the stages of assembly, the composition, stability, and dismantling of nuclear rods.

The present work investigates the formation of cytoplasmic actin-cofilin rods as a result of experimental treatments that simulate transient stress states that can still be reversed to normal physiological states. Several other studies already reported that actin-cofilin rods are formed in result of either ATP depletion or other stress conditions (Yahara et al., 1996; Minamide et al., 2000; Ashworth et al., 2003; Bernstein and Bamburg, 2003; Huang et al., 2008; Bernstein et al., 2012; Won et al., 2018). Here, we took advantage of *Dictyostelium*, an established model for cytoskeletal research, and have analyzed the composition and dynamics of cytoplasmic actin-cofilin rods and followed their formation after application of different stressors by live-cell imaging. Our data provide evidence that cytoplasmic actin-cofilin rods are formed not only in response to energy depletion, but also show that acidification of the cytoplasm is an important factor that can trigger the formation of actin-cofilin assemblies.

## Materials and Methods

### Strains and Cell Culture

All *Dictyostelium discoideum* strains used in this study derived from the axenic strain AX2-214 (considered as wild type), and were cultivated in Petri dishes in HL5 medium (Formedium) at 22C. *Dictyostelium* cells expressing GFP-cofilin or mCherry-cofilin, or the mutant strain lacking Aip1 (Konzok et al., 1999) and expressing GFP-cofilin were described earlier (Ishikawa-Ankerhold et al., 2017).

### Energy Depletion, Hyperosmotic Shock, pH Adjustment and Drug Treatment

For all experiments, *Dictyostelium* cells were cultivated in HL5 medium at 22°C. For the specific treatments, the medium was replaced by HL5 medium supplemented with the respective agent.

*Sodium azide* (*NaN*_*3*_): 10 mM in HL5 medium. To test the stability of rods when the stress stimulus is removed, cells treated with 10 mM sodium azide for 60 min were washed and incubated in HL5 medium for recovery.

*2, 4-Dinitrophenol (DNP)*: 200 µM in HL5 medium.

*Hyperosmotic shock (SOR)*: 200 and 400 mM D-sorbitol (Sigma-Aldrich) in HL5 medium.

*Sorbic acid (SA) and propionic acid (PA)*: the respective pH values of the HL5 medium were adjusted with stock solutions of 14 mM sorbic acid or 134 mM propionic acid.

*2-deoxy-glucose (2-DG)*: HL5 medium without glucose (Formedium HLB0102) supplemented with 10 g/L 2-deoxy-D-glucose (Roth)

*Cytochalasin D (CytD) and Latrunculin A (LatA)* (both Sigma-Aldrich): final concentrations of 1, 2, 4, 6, 8, 10, and 20 µM, or 1, 5, and 10 μM, respectively, in HL5 medium were tested. *Dictyostelium* cells expressing GFP-cofilin were plated on 12-mm cover glasses placed in 24-well plates and treated with cytochalasin D or latrunculin A for 60 min before induction of rod assembly with 10 mM sodium azide for 60 min. Then, cells were fixed with methanol at −20°C for 15 min and stained for actin.

The experiments were repeated at least 3 times for each treatment. For quantification, cells with cytoplasmic rods were counted from at least 10 different fields of view (image size of 512 × 512 µm).

For quantification of cells with cytoplasmic rods, Zen blue software was used (Carl Zeiss Microscopy).

### Vector for Ratiometric pH Measurements

For cytosolic pH measurements, the ratiometric fluorescent protein pHluorin2 (Mahon 2011) was cloned via *Hin*dIII and *Cla*I into a pDEX-based *Dictyostelium* vector enabling expression under control of an actin-15 promoter and conferring resistance to blasticidin (Müller-Taubenberger, 2006). The pEntry-mCherry-pHluorin2 vector (GFP-RFPpHluorin; plasmid Y872) was a kind gift of Prof. Simon Alberti (Dresden), and was used as template for amplification in combination with the primers 5′-gtg aag ctt aaa atg cca act ttg tac aaa aaa gca gg-3′ and 5′-gtg atc gat tta ttt gta tag ttc atc cat gcc atg tg-3′.

AX2 cells were transfected by electroporation using a gene pulser (Bio-Rad Laboratories) at 0.8–0.9 kV and 3 µF in 4-mm cuvettes. Transformants were selected and cultivated in the presence of 10 µg/ml blasticidin-S (ICN Biomedicals Inc.).

### Immunocytochemistry and Antibodies

Cells were plated on round 12-mm glass cover glasses coated with poly-lysine (Merck) and placed in 24-well plates, and after 1 h were subjected to the specified treatment. Thereafter, cells were fixed in methanol at −20°C for 15 min and labeled as described (Ishikawa-Ankerhold et al., 2017). After fixation, cells were washed three times with PBS, and then incubated in blocking buffer (PBS plus 2% bovine serum albumin) for 1 h at room temperature (RT). After blocking, the cells were washed three times with PBS and immunolabeled with the primary antibodies. Secondary antibodies were added for 1 h at RT. Then, samples were washed and mounted using Gelvatol or Dako. Primary antibodies used were specific for: actin (mAb Act1) (Simpson et al., 1984), Aip1 (mAb 246-153-2 and 246-404-2) (Konzok et al., 1999), coronin (mAb 176-3-6) (de Hostos et al., 1991), cofilin (pAb 496) (Ishikawa-Ankerhold et al., 2017), and isotype IgG (Molecular Probes). Secondary antibodies used in the study were Cy3, Alexa Fluor-488, -563, or -594 goat anti-mouse or anti-rabbit IgG (Molecular Probes). For some experiments, Atto 550-phalloidin (Molecular Probes, Sigma-Aldrich), and DAPI (1:1,000) were added together with the secondary antibodies.

### Confocal Microscopy

A confocal LSM 880 microscope (Carl Zeiss Microscopy) equipped with an Airyscan module and Plan-Apo 20x/NA 0.8 and Plan-Apo 63x/NA 1.46 oil immersion objectives, or a confocal LSM 510 microscope (Carl Zeiss Microscopy) equipped with a 63x Neofluar 1.4 oil immersion objective were used for image acquisition.

For live-cell microscopy, *Dictyostelium* cells expressing GFP-cofilin were plated into an open chamber or into 35-mm glass-bottom dishes (ibidi, µ-Dish 35 mm, high glass bottom), and incubated and recorded in medium or in medium adapted to the respective treatment conditions.

FRAP (fluorescence recovery after photobleaching) measurements were performed as described previously for nuclear rods (Ishikawa-Ankerhold et al., 2017). In short, living cells with cytoplasmic GFP-cofilin rods were photobleached using a LSM 510 Meta laser scanning confocal microscope with a 488-nm laser line at 100% intensity for 20 interactions as described in the detailed protocol (Müller-Taubenberger and Ishikawa-Ankerhold, 2013).

For the actin-cofilin rod disassembly experiments, the GFP-cofilin cells were plated on coverslips coated with poly-lysine, and treated with 10 mM sodium azide for 1 h at RT to induce the assembly of cytoplasmic actin-cofilin rods. After rod induction, the cells were washed to remove the chemical stimulus and incubated with medium for recovery. After 0, 5, 10, 15, 20 and 30 min under medium conditions, the cells were fixed with cold methanol (−20°C) for 15 min, washed and mounted for image acquisition.

### Ratiometric Measurements

For ratiometric pH measurements, *Dictyostelium* cells expressing GFP-mRFP-pHluorin2 were seeded at 1 × 10^4^ per ml into 35-mm glass-bottomed dishes (ibidi, µ-Dish 35 mm, high glass bottom), before imaging. The images were acquired using a LSM 880 confocal microscope with an Airyscan module (Carl Zeiss Microscopy). All imaging parameters for excitation, detection and all software settings were kept identical for the different sample acquisitions [laser intensity 488 nm (2%) and 561 nm (2%), image size (212 × 212 µm), Z-stack (30 µm), objective Plan-Apochromat ×20/0.8, pixel time (0.77 s), frame time (943.72 s)].

The images were analyzed with the ZEN software using the plugin (Histo), where a defined ROI of 4 µm^2^ was applied to the selected cells to obtain the mean fluorescence intensity from each channel (green and red). The ratios of green/red channels were plotted as median with interquartile range to represent the pH fluorescence changes inside the cells. pHluorin2 allowed us to monitor decreases of the intracellular pH after acidification of the extracellular medium, which was identified by an increase in the ratio of pH-sensitive GFP versus pH-insensitive mCherry fluorescence. In measurements where we tested the application of HL5 medium adjusted to pH values below 5.5 (pH 5.25 and 5.0), we did not detect a linear increase of the GFP fluorescence anymore, and therefore show only the measurements for pH 5.75 and 5.5.

### Statistics

Statistical analyses were calculated and graphically plotted with GraphPad Prism 9 (GraphPad Software, La Jolla, CA, United States). Data are means ± standard deviation of the mean (SD). Statistical analyses were conducted as described in the figure legends. Findings were considered statistically significant at *p* < 0.05.

## Results

### Characterization of Cytoplasmic Actin-Cofilin Rods Induced by ATP-Depletion

Earlier work conducted in our laboratory and data from other studies suggested that cytoplasmic actin-cofilin rods are induced by treatments that cause depletion of ATP (Ashworth et al., 2003; Bernstein and Bamburg, 2003; Bernstein et al., 2006; Huang et al., 2008). The current investigations aimed to identify similarities and differences between nuclear and cytoplasmic rods in *Dictyostelium* cells, and to analyze the formation of cytoplasmic rods under different stress conditions by live-cell imaging.

**FIGURE 1:**
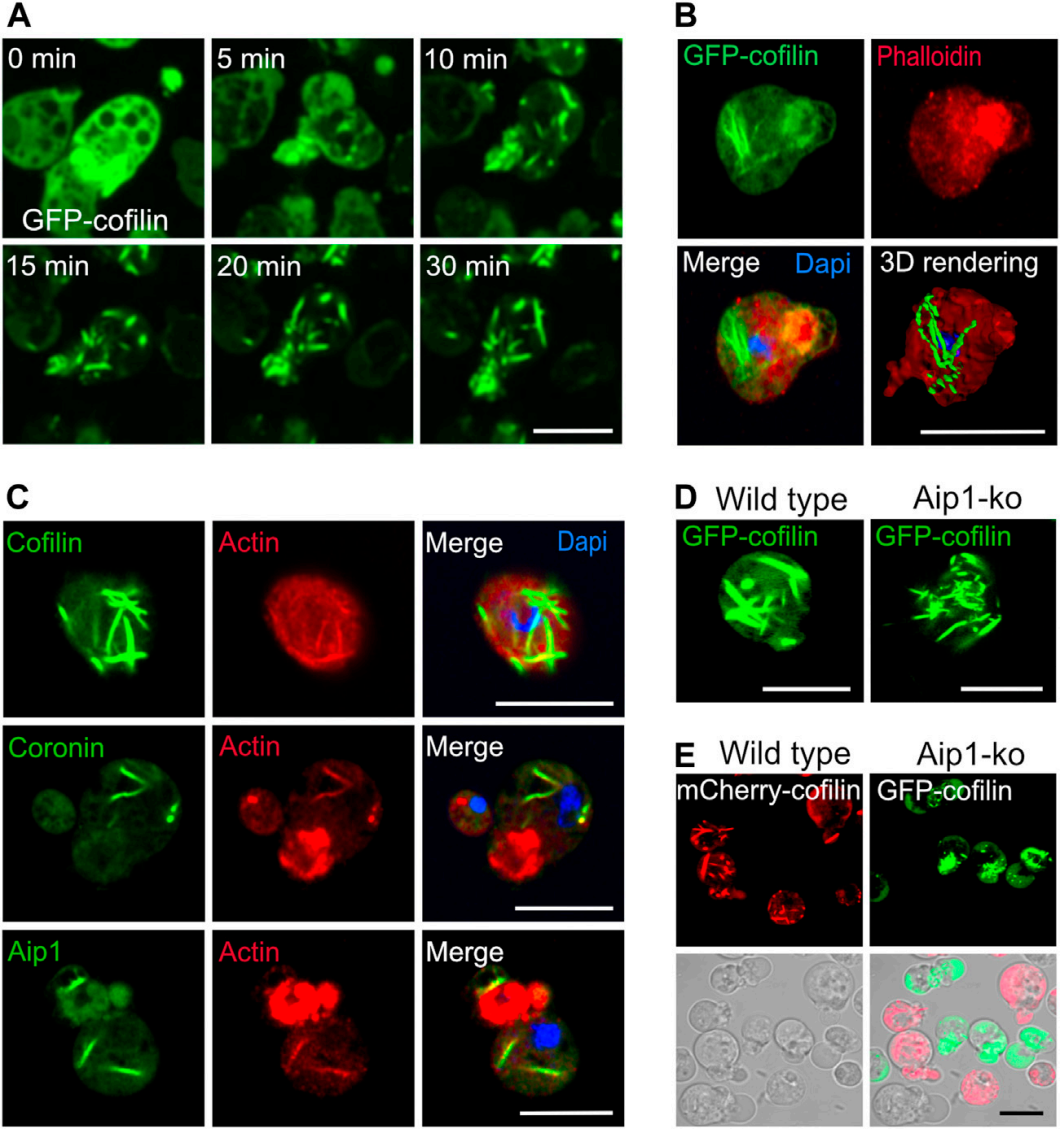
Cytoplasmic actin-cofilin rods induced by sodium azide. **(A)** Time series of confocal images of rod formation induced by 10 mM sodium azide. *Dictyostelium* cells expressing GFP-cofilin (green) were exposed to medium containing 10 mM sodium azide, and recorded by live-cell microscopy for 30 min. The time series show that already after 5 min of treatment, GFP-cofilin starts to redistribute from cortical areas into bundles of increasing size inside the cytoplasm. See also Supplementary Video S1 for the complete time series showing an overview of several cells. **(B)** Cytoplasmic actin-cofilin rods do not bind phalloidin. *Dictyostelium* cells expressing GFP-cofilin were fixed after 60 min of sodium azide treatment. GFP-cofilin (green) visualizes the cytoplasmic rods, phalloidin (red) is not co-localizing with GFP-cofilin rods. Dapi was used to label the nucleus. 3D rendering of the Z-stack projection was reconstructed using Imaris software. **(C)** Immunofluorescence labeling of cytoplasmic rods for the main constituents, actin and cofilin, as well as Aip1 and coronin after induction by sodium azide treatment in *Dictyostelium* wild-type cells. Cofilin, Aip1 and coronin were immunolabeled with the respective primary antibodies (green) and co-immunolabeled by an anti-actin antibody (red). Dapi was used to mark the nucleus (blue). Isotype controls are shown in Supplementary Figure S1A. **(D)** Rod formation after sodium azide treatment in *Dictyostelium* wild-type and Aip1-knockout (ko) cells expressing GFP-cofilin. **(E)** *Dictyostelium* cells expressing mCherry-cofilin and GFP-cofilin expressed in wild-type (red) or in Aip1-ko (green) cells were platted together and treated with sodium azide for 60 min. In the absence of Aip1, GFP-cofilin rods are not compacted into thicker bundles and remain more dispersed in comparison to wild-type cells. All scale bars, 10 µm.

In first experiments, ATP-depletion was provoked experimentally by addition of sodium azide (Ishikawa-Ankerhold et al., 2017), which acts by uncoupling the respiratory chain. Sodium azide induces cytoplasmic actin-cofilin rods both in wild-type as well as in GFP-cofilin expressing cells to a very similar extent (Supplementary Figure S1), and in order to follow formation of actin-cofilin rods by live-cell imaging, most experiments were performed with GFP-cofilin expressing cells. Addition of sodium azide to the cells causes within minutes a rapid shape change and reorganization of the actin cytoskeleton (Figure 1A and Supplementary Video S1). First actin-cofilin rods become visible within 10 min of sodium azide treatment, and after 1 h, all cells are rounded up, and rods are detected in about 80% of cells. Image reconstruction using 3D rendering confirmed the cytoplasmic localization of actin-cofilin rods (Figure 1B and Supplementary Video S2).

Our earlier studies on nuclear rods showed that these contain in addition to actin and cofilin a distinct set of other proteins comprising actin-interacting protein 1 (Aip1), coronin (CorA), filactin (Fia), and the 34-kDa actin-bundling protein B (AbpB), and we found a finely tuned spatial-temporal pattern of protein recruitment during formation of rods inside the nuclei (Ishikawa-Ankerhold et al., 2017). For cytoplasmic rods, we confirmed actin and cofilin as the main constituents (Figure 1C, Supplementary Figure S1C, and Supplementary Video S3), and by immunofluorescence labeling we identified in addition Aip1 and coronin (Figure 1C), but did not detect, in contrast to nuclear rods, a sequential recruitment.

Like for nuclear rods, the compaction of cytoplasmic rods requires Aip1, a protein that induces the disassembly of actin filaments in conjunction with cofilin (Nadkarni and Brieher, 2014). Induction of cytoplasmic rods by sodium azide in a mutant lacking Aip1 (Konzok et al., 1999) revealed that rods are less compacted (Figures 1D,E), a result suggesting that the turnover of actin is a critical determinant for unimpeded rod formation.

This assumption is further supported by the application of drugs that interfere with actin polymerization. We have used cytochalasin D (CytD) that by binding to the high affinity growing ends prevents the elongation of actin nuclei and filaments (Casella et al., 1981), and latrunculin A (LatA), a drug that binds actin monomers and thus prevents polymerization of actin (Morton et al., 2000). The formation of cytoplasmic rods was increasingly inhibited with rising concentrations of CytD (Supplementary Figure S2A). Similarly, LatA inhibited the emergence of rods in a dose-dependent manner (Supplementary Figure S2B). These results indicate that under conditions of low ATP, the turnover of actin and the availability of actin monomers are crucial and limiting for the formation of cytoplasmic rods. In contrast to intranuclear rods, cytoplasmic actin-cofilin rods are not stainable with phalloidin (Figure 1B and Supplementary Video S2), which indicates that these assemblies are structurally different to cortical actin and intranuclear rods, a finding that has been described already earlier (Nishida et al., 1987; Ishikawa-Ankerhold et al., 2017).

**FIGURE 2:**
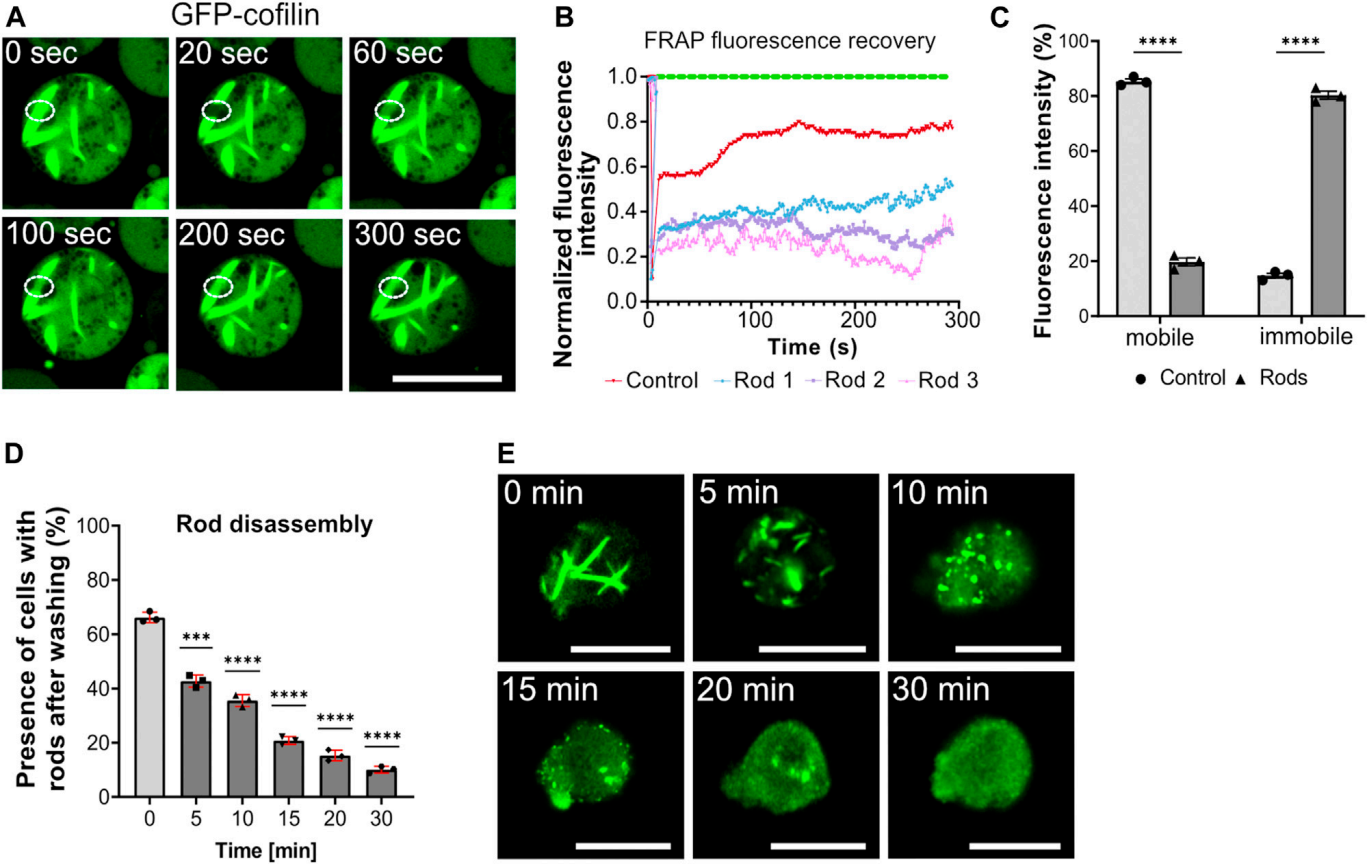
Cytoplasmic actin-cofilin rod assembly/disassembly dynamics. **(A)** Fluorescence recovery after photobleaching (FRAP) of cytoplasmic rods. Formation of cytoplasmic rods was induced by addition of 10 mM sodium azide for 60 min. Rods were selectively photobleached by drawing a ROI to apply high laser power for 10 s to bleach the GFP. The GFP diffusion into the ROI area (fluorescence recovery) was recorded for 300 s. See also Supplementary Video S4. Scale bar, 10 µm. **(B)** Fluorescence recovery of photobleached rods. Bleaching curves of three different cytoplasmic rods of sodium azide-treated cells (blue, pink, purple lines) and the untreated control cell are plotted (red). **(C)** The histogram depicts the relative proportions of mobile and immobile fractions of cells without rods (control) and with cytoplasmic rods. The average of fluorescence recovery was about 20%, indicating that rods are rather stable structures with low exchange inside the rod bundles. **(D)** Actin-cofilin rods are rapidly disintegrating structures. After induction of cytoplasmic rods by addition of 10 mM sodium azide, the percentage of cells with cytoplasmic rods decreases rapidly when the sodium azide is washed out and replaced by standard medium. Three experiments were performed and for each experiment at least 50 cells were evaluated. Data are presented as mean ± SD (red bars). Statistical significance was calculated by unpaired Welch´s t-test. *p* ≤ 0.05 was considered significant and (***p < 0.0001). **(E)** Visualization of actin-cofilin rod disassembly stages. Within 30 min after wash-out of the sodium azide, actin-cofilin rods are almost completely disintegrated. The disassembly takes place via shorter bundles and punctiform actin-cofilin aggregates. Scale bars, 10 µm.

In order to address the change in the dynamics of cytoplasmic actin-cofilin rods over time (i.e., newly assembled versus “aged” rods), we analyzed the exchange between the assembled and diffuse pool of GFP-cofilin by FRAP (fluorescence recovery after photobleaching). For this, GFP-labeled rods were bleached using high laser power (100%), and fluorescence recovery was monitored over time (Figures 2A–C and Supplementary Video S4). The mobile fraction represented by the fluorescence recovery of GFP-cofilin rods after photobleaching was about 20%, meaning that cytoplasmic rods are rather stable structures with low protein exchange.

The formation of actin-cofilin rods is rapidly reversible when the stress stimulus is removed (Figures 2D,E). After induction of cytoplasmic rods by addition of 10 mM sodium azide, the percentage of cells with cytoplasmic rods decreased rapidly when the sodium azide was washed out and replaced by standard HL5 medium. 5 min after replacement of sodium azide by medium, cytoplasmic rods started to disassemble, recognizable as shortening cofilin bundles. In the subsequent recovery phase, the bundles were shortened further and appeared as punctuated small cofilin aggregates, which continued to disintegrate.

Another drug that causes energy depletion is 2,4-dinitrophenol (DNP). DNP acts as protonophore and causes uncoupling of respiratory chain oxidative phosphorylation thereby inhibiting ATP production. Previous work by Gerisch and colleagues (Jungbluth et al., 1994) showed that addition of 50 µM DNP causes the reversible compartmentalization of the actin cytoskeleton. However, the available microscopical techniques were limited at that time, and did not allow a detailed resolution of cytoskeletal structures.

**FIGURE 3:**
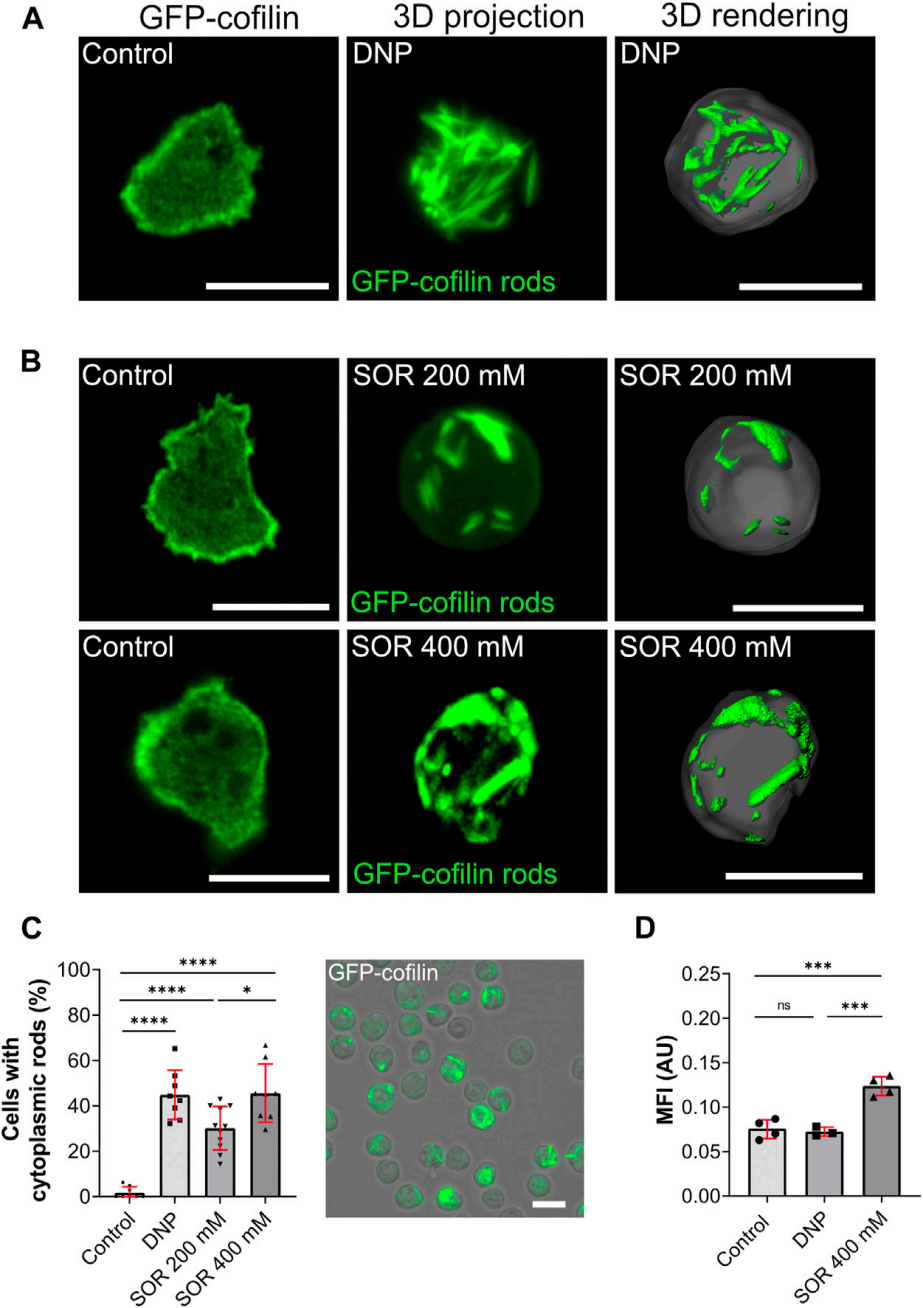
Cytoplasmic actin-cofilin rods induced by DNP or hyperosmotic shock. **(A)** Non-treated cells (vehicle control), 3D projection and image rendering of rods formed by GFP-cofilin expressing cells treated with 200 µM DNP for 2 h. Scale bar, 10 µm. **(B)** Fluorescent images of non-treated GFP-cofilin expressing cells (vehicle control) and cytoplasmic actin-cofilin rods induced by treatment with 200 or 400 mM sorbitol (SOR). Scale bar, 10 µm. **(C)** Percentage of cells with rods after treatment with DNP or sorbitol for 2 h. Fluorescence image on the right shows a representative overview used for quantification (212 × 212 µm). For each experiment (*n* > 6), 10x overview images were quantified. Scale bar, 10 µm. **(D)** Ratiometric fluorescence measurements show a drop in the intracellular pH after treatment with 400 mM sorbitol for 2 h. Histograms depict median fluorescence intensity (MFI). More acidic values result in higher MFI. Error bars are median with interquartile range (red). Samples were considered significant when *p* ≤ 0.05. Welch´s t-test was applied. *n* = 3-4 experiments; more than 20 cells were analyzed per experiment, and the average of fluorescence intensity was determined.

We have visualized the formation of cytoplasmic actin-cofilin rods in GFP-cofilin expressing cells after addition of DNP (Figure 3A). Live-cell microscopy revealed a rapid reorganization of the actin cytoskeleton after addition of DNP, and rods became visible within 10–15 min (Supplementary Figure S3A). After 2 h with DNP, 50% of cells showed cytoplasmic rods. Rod formation was fully reversible after an incubation time of 3 h by replacing the DNP medium with standard medium similar to the recovery after sodium azide treatment.

### Formation of Cytoplasmic Actin-Cofilin Rods Under Hyperosmotic Conditions

Next, we tested hyperosmotic stress as another condition that we assumed to potentially induce cytoplasmic actin-cofilin rods. Hyperosmotic stress was induced by addition of 200 and 400 mM sorbitol (Figure 3B). It was reported earlier that in consequence of hyperosmotic conditions, *Dictyostelium* cells rearrange their cytoskeleton. This transition was postulated to constitute the major osmoprotective mechanism in *Dictyostelium* accompanied by a rapid acidification of the cytosol (Ott et al., 2000; Pintsch et al., 2001), but the cytoskeletal changes were not investigated in greater detail. In addition, it was found that vesicle mobility and membrane flow were downregulated under hyperosmotic conditions (Pintsch et al., 2001) a notion supported in studies of fluid- to solid-like state transitions more recently (Munder et al., 2016). We found that hyperosmotic conditions caused a rapid formation of cytoplasmic actin-cofilin rods (Figure 3B). After 2 h of incubation with 200 mM sorbitol, about one third of the cells showed cytoplasmic rods, with 400 mM sorbitol about 50% (Figure 3C).

Since hyperosmotic conditions were described to be accompanied by a rapid drop in intracellular pH, we next set out to determine changes in the intracellular pH of *Dictyostelium* cells. Previous studies using ^31^P-NMR (nuclear magnetic resonance) showed that the intracellular pH of growth-phase *Dictyostelium* cells is around pH 7.3 (Kay et al., 1986). To directly monitor changes of the intracellular pH, we initially tested established intracellular pH indicators used for pH determination in other eukaryotic cell lines. Under our experimental conditions, BCECF (Invitrogen) and pHrodo (Life technologies) were not usable because they were rapidly sequestered into the endosomal compartment of *Dictyostelium* cells. We therefore generated a vector for expression of the ratiometric pH sensor pHluorin2 (Mahon, 2011), and examined pHluorin2 expressing *Dictyostelium* cells in live-cell measurements in order to monitor changes in the intracellular pH after reduction of the pH of the extracellular medium to 5.75 and 5.5. While DNP had no effect on the intracellular pH of *Dictyostelium* cells, application of 400 mM sorbitol caused a reduction of the cytoplasmic pH (Figure 3D) consistent with earlier results (Pintsch et al., 2001).

### Reduction of Cellular pH Induces the Formation of Cytoplasmic Actin-Cofilin Rods

**FIGURE 4:**
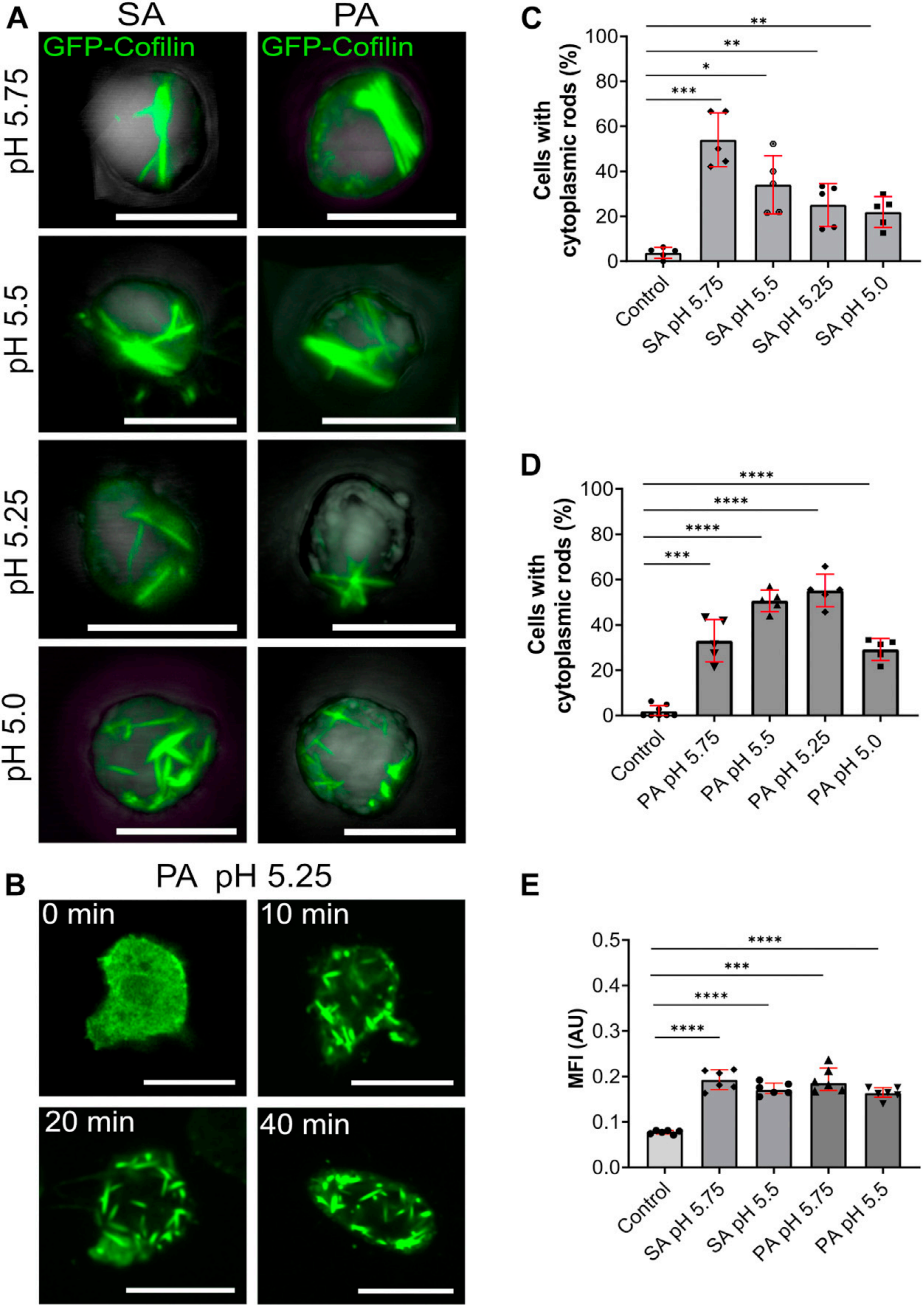
Cytoplasmic actin-cofilin rods are induced by lowering the intracellular pH. **(A)** 3D projection images of GFP-cofilin rods (green) formed by GFP-cofilin expressing cells cultivated for 2 h in medium adjusted to lower pH values with sorbic acid (SA) or propionic acid (PA) (pH 5.75, 5.5, 5.25 and 5.0). Scale bars are 10 µm. **(B)** Time lapse images of GFP-cofilin expressing cells placed in medium adjusted to pH 5.25 with PA. After 10 min, GFP-cofilin rods (green) start to assemble in the cytoplasm. Scale bars, 10 µm. **(C,D)** Percentage of cells with GFP-cofilin rods in medium adjusted with SA **(C)** or PA **(D)** to the indicated pH values. *n* = 5 experiments. **(E)** Ratiometric pH measurements to determine acidification of the intracellular pH. *Dictyostelium* cells expressing pHluorin2 cells were incubated with medium adjusted with either SA or PA to pH 5.75 and 5.5. An increase of MFI indicates the lowering of the intracellular pH. *n* = 6 experiments per treatment. More than 20 cells were analyzed for each experiment and treatment. Error bars are median with interquartile range (red). Samples were considered significant when *p* ≤ 0.05. Welch´s t-test was applied.

In order to investigate whether an acidification of the cytosol itself could trigger the formation of cytoplasmic actin-cofilin rods, we systematically examined the formation of rods by increasingly lowering the pH of the extracellular medium by the addition of weak acids. Under standard laboratory conditions, axenically growing *Dictyostelium* cells are cultivated in medium with a pH of 6.4. By addition of weak membrane-permeable acids, sorbic or propionic acid, we reduced the pH of the growth medium (5.75, 5.5, 5.25, 5.0) and followed the formation of cytoplasmic actin-cofilin rods either in fixed cells (Figure 4A) or by live-cell microscopy (Figure 4B). All pH conditions tested provoked the formation of cytoplasmic actin-cofilin rods. The rods varied from bar-shaped at low extracellular pH (pH 5.75 and 5.5) to more needle-like condensates at very low extracellular pH conditions (pH 5.25 and 5.0) (Figures 4A,B and Supplementary Figure S3B). With sorbic acid buffered medium, the percentage of cells with rods was highest with the slightest pH-shift (pH 5.75), and then decreased at lower values (Figure 4C). With propionic acid buffered medium, about 60% of cells formed cytoplasmic rods at pH 5.25, but rods were also detected at a considerable extent at the other pH values tested (Figure 4D). Ratiometric measurements of cells in sorbic and propionic acid containing medium confirmed the reduction of the intracellular pH compared to control cells (Figure 4E).

### Starvation by Glucose Deprivation Induces Development and is Accompanied by a Transient Appearance of Cytoplasmic Rods

The life cycle of *Dictyostelium* cells is characterized by two main phases, the growth stage, in which the cells multiply by mitotic divisions, and a developmental stage, in which the cells aggregate to form a multicellular slug and finally fruiting bodies. The transition from growth to development is induced by starvation.

**FIGURE 5:**
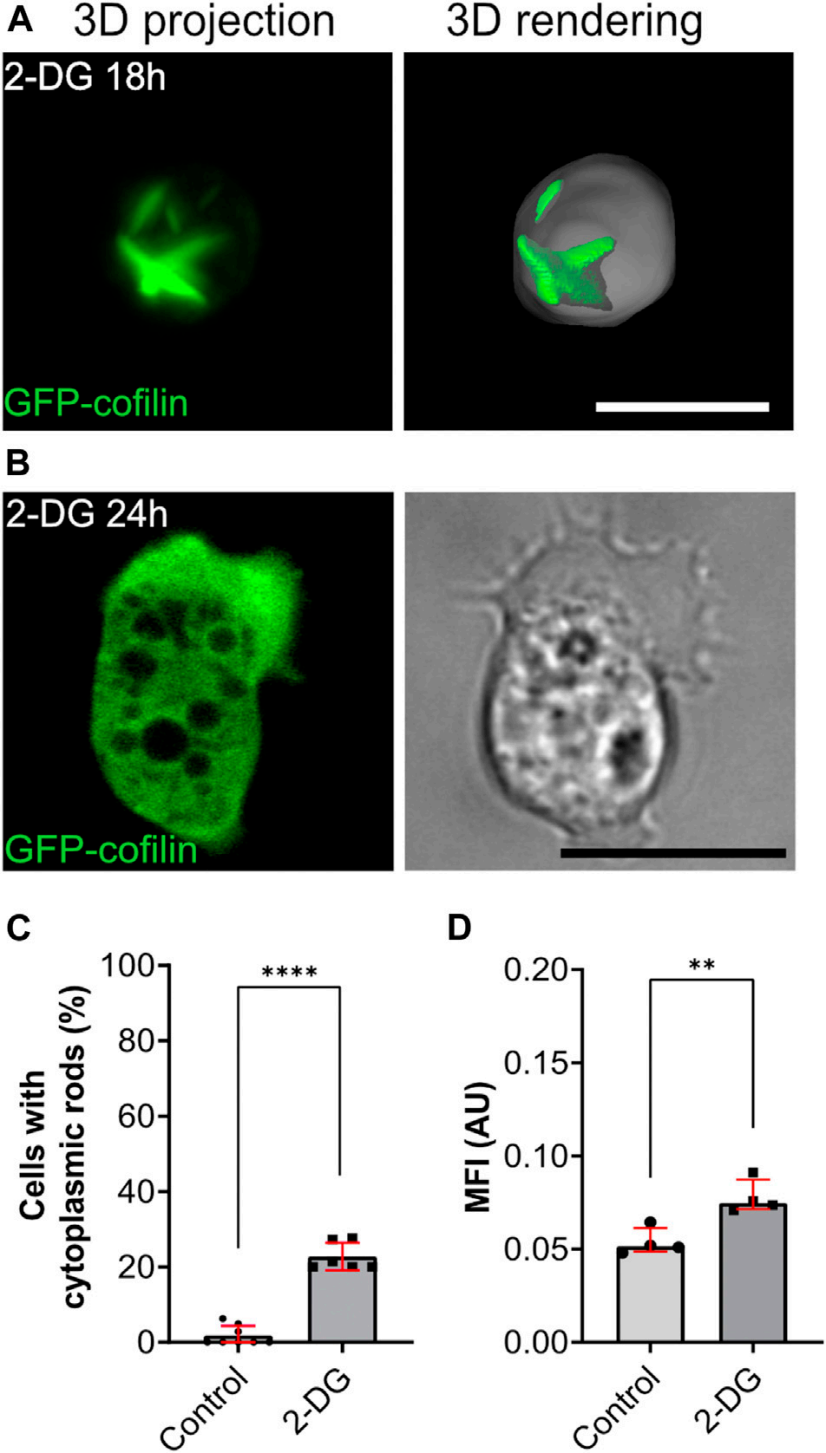
Starvation induced by glucose-deprivation (2-DG) causes transient formation of cytoplasmic actin-cofilin rods. **(A)** 3D projection and rendering of GFP-cofilin expressing cells treated with 2-DG for 18 h, or **(B)** 24 h. Scale bars, 10 µm. **(C)** Percentage of cells with cytoplasmic rods after 18 h of glucose deprivation. For each experiment, 10x images (212 × 212 µm) were used for quantification (*n* = 6). **(D)** Ratiometric pH measurements 18 h after incubation of cells in 2-DG-containing medium. For each experiment, 10x images (212 × 212 µm) with a total of more than 20 cells per experiment were analyzed to determine the mean of fluorescence intensity (*n* = 4). Error bars are median with interquartile range (red). Samples were considered significant when *p* ≤ 0.05. Welch´s t-test was applied.

In the course of experiments where we tested energy depletion, we cultivated *Dictyostelium* cells in glucose-free medium containing 2-deoxyglucose (2-DG). In contrast to mammalian cells, application of 2-DG did not result in the same effect as ATP depletion with sodium azide or DNP. Instead, we observed that after 5–6 h a low percentage of cells (<5%) were characterized by the appearance of rods. After 18 h, about 20% of cells showed cytoplasmic rods (Figures 5A,C). Ratiometric measurements of pHluorin-cells after application of 2-DG medium revealed a significant drop in the intracellular pH after 18 h (Figure 5D). After 24 h, rods were not detectable anymore, but most cells were characterized by an elongated shape typical for *Dictyostelium* cells of the aggregation stage (Figure 5B).

## Discussion

A series of studies suggested that under conditions that cause cellular stress, complexes of actin and cofilin, so-called actin-cofilin rods, are formed and interfere with essential cell functions, and consequently were held responsible for causing the disease states (Bamburg and Wiggan, 2002; Maloney and Bamburg, 2007; Bamburg et al., 2010; Munsie et al., 2011; Hoffmann et al., 2019; Wang et al., 2020). Experimental work also showed that formation of actin-cofilin rods can be provoked by various types of stressors either inside the nucleus or in the cytoplasm. However, despite the finding that the appearance of actin-cofilin rods is linked to several diseases, fundamentally important questions remained unanswered. For instance, it is unknown whether these structures are related, similar in their composition, or serve a specific function to maintain the cellular equilibrium. Similarly, the functional differences of nuclear and cytoplasmic rods are unclear. The current picture is that the formation of actin-cofilin rods is much more complex and may be triggered by different causes.

Even though the exact role of actin-cofilin rods still needs to be elucidated in more detail, the general assumption is that the formation of rods constitutes a strategy for the cell to reduce energy consumption due to a shut-off of actin-treadmilling. The constant turnover of actin is a highly energy-dependent process, which consumes up to 50% of the cellular ATP (Daniel et al., 1986; Bernstein and Bamburg, 2003). Thus, actin-cofilin rod formation may provide a protective mechanism for cells under stress conditions. This notion is supported by the assumption that cellular metabolism and actin remodeling are coupled processes (DeWane et al., 2021).

In the present study we have used the *Dictyostelium* model system to explore the characteristics and principles of cytoplasmic actin-cofilin rod formation. Like nuclear rods, cytoplasmic rods consist of actin and cofilin and contain coronin and Aip1 as associated proteins. However, we do not find the finely tuned spatial-temporal pattern of protein recruitment that we found for nuclear rod assembly (Ishikawa-Ankerhold et al., 2017). Conditions that cause depletion of ATP, like blockage of the respiratory chain, induce the formation of cytoplasmic actin-cofilin rods. Limiting ATP production is accompanied by the production of reactive oxygen species (ROS), and several studies indicated that ROS induce the formation of actin-cofilin rods (reviewed by Bamburg and Bernstein, 2016). Though we were not able to distinguish whether ATP depletion or ROS formation is decisive for the formation of cytoplasmic rods, in our initial experiments we have applied sodium azide known to block the mitochondrial respiratory chain, and followed and visualized rod formation by live-cell imaging.

In contrast to nuclear rods, cytoplasmic rods are not stainable by phalloidin suggesting a variant arrangement of filamentous actin in the rods (Nishida et al., 1987; Aizawa et al., 1999; Ishikawa-Ankerhold et al., 2017). Similar to nuclear rods, Aip1 is a crucial factor for the compaction of cytoplasmic rods. Mutant cells lacking Aip1 are characterized by less compacted and more needle-like cytoplasmic rods. Aip1, as well as coronin, have been shown to cooperate with cofilin to enhance depolymerization of filamentous actin (Ono, 2003; Kueh et al., 2008; Ishikawa-Ankerhold et al., 2010; Nadkarni and Brieher, 2014; Mikati et al., 2015).

Application of cytochalasin D, a drug blocking the fast-growing ends of actin filaments, inhibits the assembly of cytoplasmic actin-cofilin rods already at low doses. Similarly, LatA inhibits cytoplasmic rod formation to a considerable extent. These results suggest that the availability of actin monomers as well as free barbed end are important for the formation of cytoplasmic actin-cofilin assemblies. It is likely that cofilin and cytochalasin D interfere at barbed ends, an assumption that is fostered by recent *in-vitro* data showing cofilin accelerates actin dynamics at both ends (Wioland et al., 2017; Romet-Lemonne and Jegou, 2021). From this it can be concluded that in addition to regulatory proteins such as Aip1 and coronin, the complex regulation of cofilin itself is one of the decisive factors for the formation of cytoplasmic actin-cofilin rods.

In the current study, we show that a reduction of the intracellular pH induces the assembly of actin-cofilin rods in the cytoplasm. The application of the weak acids sorbic (SA) and propionic acid (PA) to the extracellular medium, shows slight differences which may be attributed to differences in the efficiency of their membrane permeability. However, our results using the pHluorin2 as reporter show that cultivation of *Dictyostelium* cells in medium adjusted to lower pH values by either sorbic or propionic acid reduces the intracellular pH to a considerable extent. Our data are also consistent with earlier NMR-measurements that showed a reduction of intracellular pH by the addition of propionic acid (Satre et al., 1989).

We conclude that a decrease in cellular pH is a critical determinant of cytoplasmic actin-cofilin rod formation. This assumption is strengthened by the finding that hyperosmotic conditions, accompanied by a drop in cellular pH, induce the formation of cytoplasmic actin-cofilin rods. Moreover, it has been shown that in *Dictyostelium* the switch between growth and development, which is induced by starvation of the cells is accompanied by a transient drop in the intracellular pH (Aerts et al., 1987). This may explain the temporary appearance of rods in our experiments employing 2-DG to simulate glucose deprivation. These results suggest that acidification or even a locally limited drop in the intracellular pH can cause the formation of actin-cofilin rod structures, and it is obvious to speculate that local changes in intracellular pH are also important determinants for the transient formation of rods in other cell types and in particular neuronal cells.

Protein aggregates and macromolecular assemblies have been shown to play a role in the development of a number of degenerative diseases, for instance neurofibrillary tangles typically associated with Alzheimer’s disease or aberrant RNP granules with ALS or FTD (frontotemporal dementia). Recent work suggested that impairments in the control of phase separation are causative for at least some neurological disease pathologies (Alberti and Hyman, 2016; Hofweber et al., 2018; Alberti and Hyman, 2021). Phase separation describes the separation of a homogenous mix of proteins into two coexisting phases, a more liquid phase that is enriched for these molecules and a phase that is depleted, and thereby enables the formation of membrane-less cellular compartments with different physical properties by the condensation of macromolecules (Alberti, 2017). How phase transitions organize cellular condensates is currently a matter of intense research (Boeynaems et al., 2018).

A current working concept is that protein phase separation may be used by cells to regulate protein synthesis and to ensure adaptation to a broad range of stress situations (Kroschwald and Alberti, 2017). De-mixing and the formation of protein assemblies may provide an evolutionarily conserved mechanism for cells to adapt to and to survive extreme situations. Our results showing that the intracellular pH could be a critical determinator for actin-cofilin rod formation fits into the recent concept that pH could be a regulator of phase separation (Kroschwald and Alberti, 2017). Whether these considerations play a role for the pathological states of neuronal cells through the formation of actin-cofilin rods needs to be examined more closely.

## Data Availability

The raw data supporting the conclusion of this article will be made available by the authors, without undue reservation.
