# Supplementary material for: Formation of Cytoplasmic Actin-Cofilin Rods is Triggered by Metabolic Stress and Changes in Cellular pH
Source: Front Cell Dev Biol. 2021 Nov 17;9:742310. doi: 10.3389/fcell.2021.742310 (PMC8635511; doi:10.3389/fcell.2021.742310)
Supplement: Supplementary file 5 [file DataSheet1.docx]

Supplementary Material

# Supplementary Figures


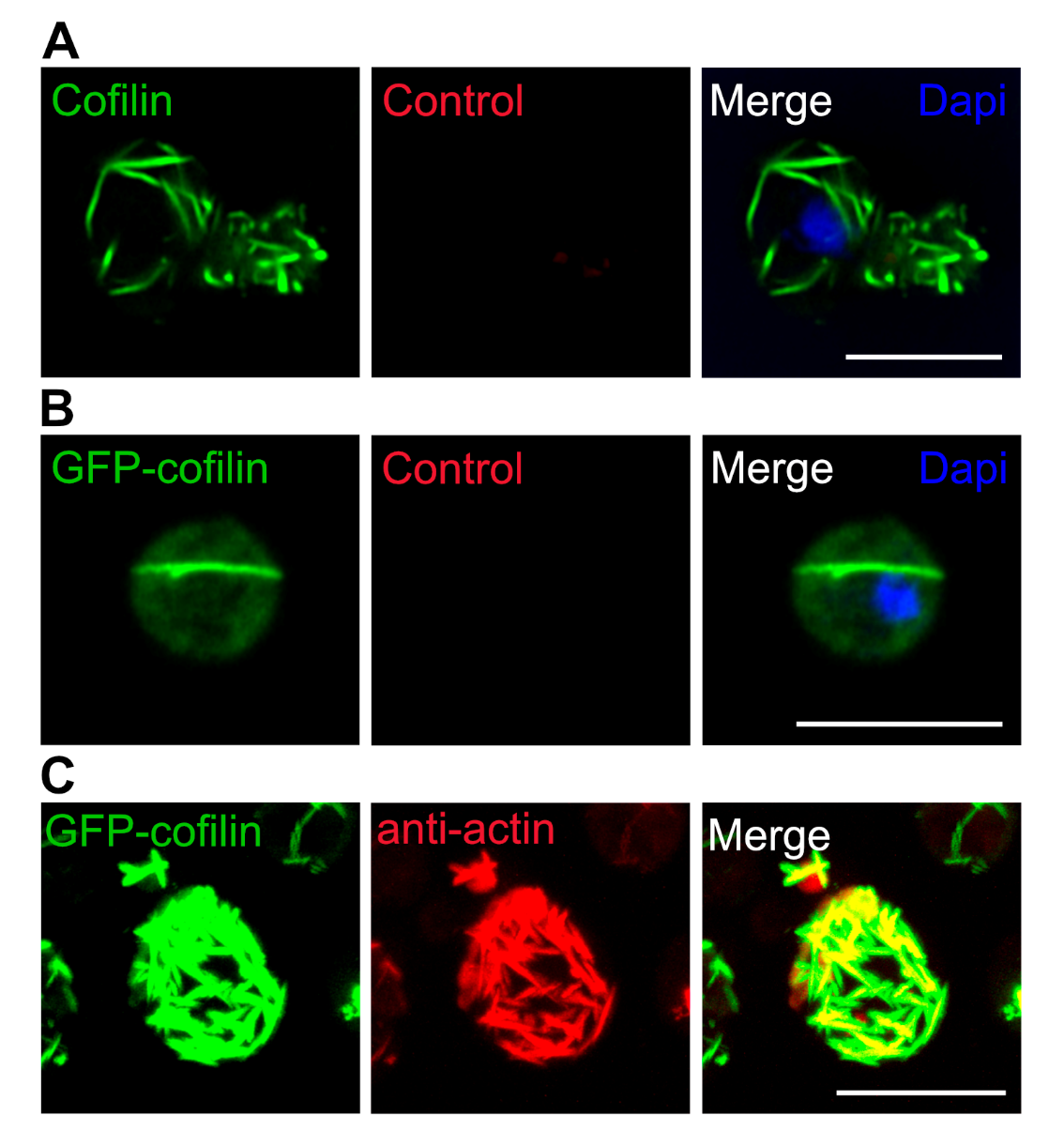


**Supplementary Figure S1. Actin and cofilin are the core proteins of cytoplasmic rods. (A)** Wild-type *Dictyostelium* cells were treated with 10 mM sodium azide for 1 h to induce cytoplasmic actin-cofilin rods. Cells were then fixed, and immunolabeled with anti-cofilin primary antibodies and secondary Alexa Fluor-488 goat anti-rabbit IgG (green). As a control for actin staining (compare Figure 1C), primary isotype IgG antibody and secondary Cy3-goat-anti-mouse antibody was used (red). Nuclear DNA was stained with DAPI (blue). (**B**) Cytoplasmic actin rod formation was induced in GFP-cofilin expressing *Dictyostelium* cells (green) by treatment with 10 mM sodium azide. Cells were fixed and stained with DAPI to visualize nuclei, and isotype IgG as control for actin-staining as in (A), or (**C**) with anti-actin antibodies (red). Secondary Cy3-goat anti-mouse secondary antibodies was applied. Please note the almost complete overlap (yellow) of green and red channels in (C). Scale bars, 10 µm.


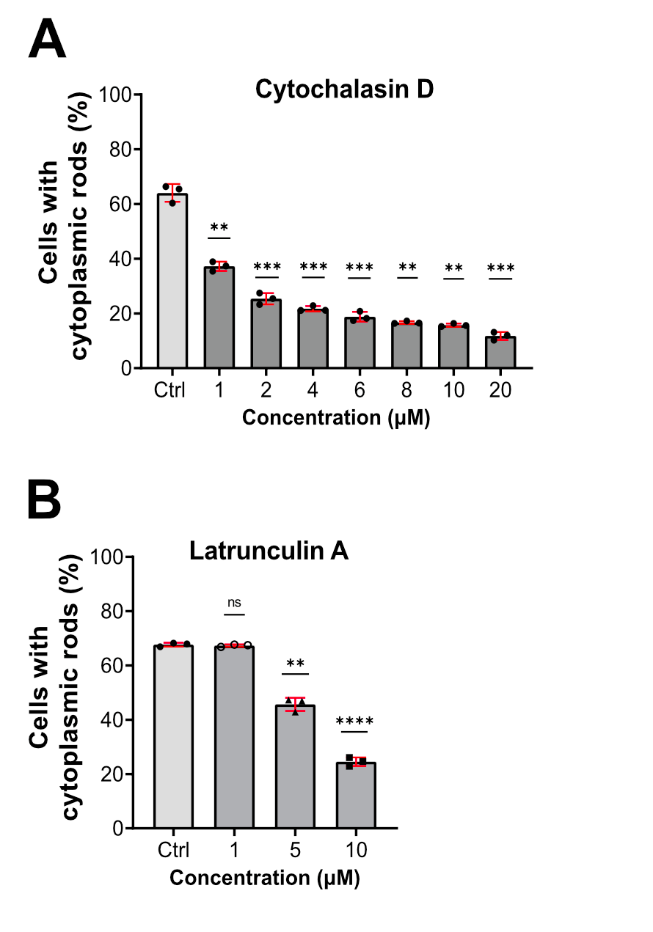


**Supplementary Figure S2. Inhibitors of actin polymerization interfere with cytoplasmic actin-cofilin rod formation.** (**A**) Cytochalasin D inhibits cytoplasmic rod assembly. GFP-cofilin expressing cells were pre-incubated with different concentrations of cytochalasin D, before actin rods were induced by addition of sodium azide. Then, cells were fixed and stained with anti-actin antibodies to visualize and quantify the number of cells containing actin-cofilin rods. (**B**) Latrunculin A affects cytoplasmic rod assembly. GFP-cofilin expressing cells were pre-incubated with different concentrations of latrunculin A, before actin rod induction with sodium azide, and further treated and evaluated as in (A). The experiments were repeated at least three times for each treatment and at least 200 cells were counted from 10 different fields of view. Data are presented as mean ± SD (red bars). Statistical significance was calculated by unpaired Welch´s t-test. p ≤ 0.05 was considered significant.

**
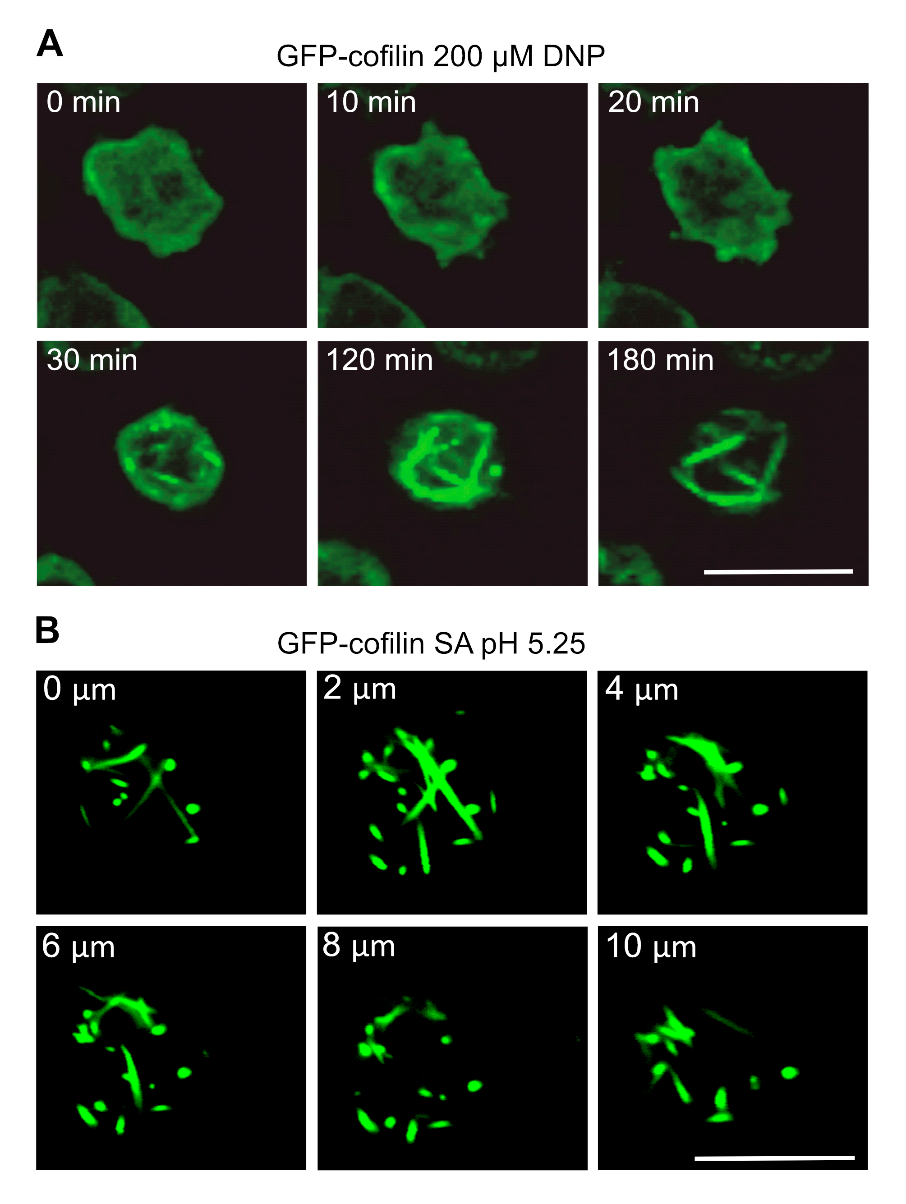
**

**Supplementary Figure S3. Cytoplasmic actin-cofilin rods induced by DNP or reduction of the intracellular pH.** (**A**) Time-lapse confocal images of GFP-cofilin expressing cells treated with 200 µM DNP were recorded for 180 min. Cytoplasmic rods start to assemble in small short bundles within 30 min of induction. After 180 min, rods have compacted into thicker long bundles. (**B**) Z-stack images of a GFP-cofilin expressing cell incubated in sorbic acid (SA)-buffered medium (pH 5.25) for 2 h. The individual layers (Z-stacks) of the imaged cell are displayed with a step size of 2 µm depicting rods in different orientations inside the cell. Scale bars, 10 µm.

# Supplementary Movies

**Movie 1. Cytoplasmic actin-cofilin rod formation in cells expressing GFP-cofilin.** Live-cell imaging of *Dictyostelium* cells expressing GFP-cofilin. Cytoplasmic rod formation was induced by treatment with 10 mM sodium azide. The time series was recorded by confocal microscopy for 60 min. Time interval is 1 sec/frame.

**Movie 2. Cytoplasmic actin-cofilin rods do not bind phalloidin**. 3D projection imaging of rods induced by 10 mM sodium azide for 60 min in GFP-cofilin (green) expressing *Dictyostelium* cells. Cells were fixed and labelled with Alexa 594-phalloidin (red) and DAPI (blue).

**Movie 3.**  **3D reconstruction** **of cytoplasmic actin-cofilin rods.** Cytoplasmic actin-cofilin rod formation was induced in GFP-cofilin expressing cells (green) by treatment with 10 mM sodium azide. Cells were fixed and labeled with anti-actin antibodies (red). Please note the almost complete overlap (yellow) of green and red channels showing the co-localization of actin and cofilin in cytoplasmic actin-cofilin bundles.

**Movie 4. Fluorescence recovery after photobleaching**. Cytoplasmic actin-cofilin rods were induced by 10 mM sodium azide for 60 min. The cytoplasmic rod was photobleached in the ROI area and the recovery of the GFP-fluorescence was monitored.
